# Alpha-fetoprotein normalization as a prognostic surrogate in small hepatocellular carcinoma after stereotactic body radiotherapy: a propensity score matching analysis

**DOI:** 10.1186/s12885-015-2017-z

**Published:** 2015-12-18

**Authors:** Jinhong Jung, Sang Min Yoon, Seungbong Han, Ju Hyun Shim, Kang Mo Kim, Young-Suk Lim, Han Chu Lee, So Yeon Kim, Jin-hong Park, Jong Hoon Kim

**Affiliations:** 1 Department of Radiation Oncology, Asan Medical Center, University of Ulsan College of Medicine, 88, Olympic-ro 43-gil, Songpa-gu, Seoul Republic of Korea; 2 Department of Biostatistics, Asan Medical Center, University of Ulsan College of Medicine, 88, Olympic-ro 43-gil, Songpa-gu, Seoul Republic of Korea; 3 Department of Gastroenterology, Asan Medical Center, University of Ulsan College of Medicine, 88, Olympic-ro 43-gil, Songpa-gu, Seoul Republic of Korea; 4 Department of Radiology, Asan Medical Center, University of Ulsan College of Medicine, 88, Olympic-ro 43-gil, Songpa-gu, Seoul Republic of Korea; 5 Asan Liver Center, Asan Medical Center, University of Ulsan College of Medicine, 88, Olympic-ro 43-gil, Songpa-gu, Seoul Republic of Korea; 6 Present address: Department of Radiation Oncology, Kyung Hee University Medical Center, Kyung Hee University School of Medicine, Songpa-gu, Seoul Republic of Korea

**Keywords:** Hepatocellular carcinoma, Stereotactic body radiotherapy, Alpha-fetoprotein, Prognostic factor

## Abstract

**Background:**

To assess the significance of alpha-fetoprotein (AFP) normalization as a prognostic surrogate after stereotactic body radiotherapy (SBRT) for patients with small hepatocellular carcinoma (HCC).

**Methods:**

Patients who underwent SBRT for primary or recurrent HCC were registered and a database thereof was retrospectively reviewed. Among 165 total registered patients, 77 patients were selected who satisfied the following criteria: (1) their AFP levels were > 20 ng/mL before SBRT, and (2) their AFP levels were checked within three months after SBRT. Propensity score based matching analysis was performed to minimize potential confounding bias. AFP normalization was defined as a reduction of AFP level to ≤ 20 ng/mL. Overall survival (OS) and progression-free survival (PFS) curves were estimated by the Kaplan-Meier method.

**Results:**

Thirty-seven (48.1 %) patients displayed AFP normalization (normalized group), while 40 (51.9 %) patients comprised the non-normalized group. The OS rates at 3-year were 62.0 % and 44.0 % (*p* = 0.023), and the PFS rates at 3-year were 27.9 % and 12.0 % (*p* = 0.019), in the normalized and non-normalized groups, respectively. Local control rates were 97.2 % in the normalized group and 94.7 % in the non-normalized group at three years (*p* = 0.579). In the propensity score matching cohort (25 pairs), OS and PFS were significantly longer in the normalized group than in the non-normalized group (*p* = 0.017 and 0.049, respectively). The local control rates were 100 % in both matched groups.

**Conclusions:**

AFP normalization within three months after SBRT is a prognostic surrogate for OS and PFS in patients with small HCC. AFP monitoring should be considered a useful tool for HCC patients with an elevated AFP level before SBRT.

## Background

Surgical resection and liver transplantation are curative treatment modalities for HCC. Surgical resection has shown overall survival (OS) and recurrence-free survival rates of 75–85 % and 83–92 %, respectively, at 3–4 years [1, 2]. Liver transplantation has shown a five-year OS rate of 70 %, as well as a low recurrence rate [1, 3]. However, only a small proportion of patients can be treated using these modalities for various clinical reasons. Another curative treatment of HCC is radiofrequency ablation (RFA), which yields a necrosis rate of 90–100 % for tumors < 2 cm in diameter and 50 % for those with a diameter > 3 cm [4, 5]. However, the proximity of the tumor to major vessels, gall bladder, diaphragm, and liver surface, as well as the presence of non-echogenic lesions on ultrasound, present major restrictions regarding the use of RFA. Consequently, overall, only 30–40 % of patients may benefit from the various curative treatments, due to various clinical conditions [6]. Therefore, an alternative local therapeutic modality, namely stereotactic radiotherapy, is urgently needed in these clinical settings.

With the development of 3-dimensional conformal radiotherapy, image-guided treatment, and the resulting accumulation of knowledge on partial-volume liver tolerance, high-dose radiation can now be delivered to focal liver volumes [7]. Hence, the use of radiotherapy for small intrahepatic tumors has become feasible as well as tolerable for patients. Several studies, including prospective and retrospective ones, have demonstrated that stereotactic body radiotherapy (SBRT) can achieve high local control and competent OS rates of 65–100 % and 48–82 %, respectively, at 1 year [8–12].

The tumor marker, alpha-fetoprotein (AFP), is detected in approximately 39–65 % of HCC patients, and has been used as a diagnostic tool [13]. Measurement of AFP is useful for detecting recurrence and for predicting survival after locoregional or systemic treatment, based on the hypothesis of the correlation between AFP level and tumor activity or tumor burden [14–17]. Although some recent studies have reported the association between AFP level and local tumor control or tumor response after SBRT [18, 19], the significance of AFP normalization has yet not been studied in HCC patients after SBRT, which is an alternative ablative treatment used worldwide for small HCC. Accordingly, this study undertook to assess the significance of AFP normalization after SBRT as a prognostic surrogate for patients with small HCC.

## Methods

### Patient selection

Patients who underwent SBRT for primary or recurrent HCC were prospectively registered at our institution, and the database of their records between March 2007 and December 2011 was retrospectively reviewed. The detailed eligibility criteria for SBRT were described in our previous report [12]. Among 165 total registered patients, 77 were selected, as they met the following criteria: (1) their AFP levels were > 20 ng/mL before SBRT, and (2) their AFP levels were checked within three months after SBRT. This study was approved by the Institutional Review Board of the Asan Medical Center, and written informed consent was waived because of the retrospective nature of the study.

### SBRT procedure

The SBRT procedure at our institution was described in our previous study [12]. At least one week before computed tomography (CT) simulation, three fiducial markers (Standard Gold Soft Tissue Markers, CIVCO Medical Solutions, Kalona, IA) were inserted into the liver parenchyma around the tumors of all patients except for those who had surgical clips or compact iodized oil from previous treatments. Pillows and vacuum molds were used for patient immobilization: 4-dimensional CT simulation was carried out (GE LightSpeed RT 16; GE Healthcare, Waukesha, WI, USA) with free breathing. The CT series were sorted according to respiratory phase using 4D imaging software (Advantage 4D version 4.2; GE Healthcare). Gross tumor volume (GTV) was delineated based on visible gross tumor seen on CT images at the end-expiratory phase; extension based on movement within the gating phase (30–70 %) from the GTV was set as the internal target volume (ITV). Planning target volume margin was 5 mm from the ITV. SBRT was planned using multiple coplanar and non-coplanar beams with energies of 6 or 15 MV. A dose of 10–20 Gy (median, 15 Gy) per fraction was given over 3–4 consecutive days to a total dose of 30–60 Gy (median, 45 Gy). Contouring and treatment planning were done using a 3D-radiotherapy planning system (Eclipse version 8.0; Varian Medical Systems, Inc., Palo Alto, CA, USA).

Image guidance, including cone-beam CT and gated fluoroscopy, was performed before each treatment session using the On-Board Imager (Varian Medical Systems).

### AFP evaluation and assessment of treatment response

Serum AFP levels were measured by chemiluminescent microparticle immunoassay (ARCHITECT i2000SR; Abbott, Chicago, IL, USA). AFP normalization was defined as the reduction of AFP to our institutional normal level (≤ 20 ng/mL) within three months after completion of SBRT. After treatment, patients were followed up every 1–3 months. Follow-up consisted of physical examinations, complete blood counts, biochemical profiles, AFP, and dynamic CT or magnetic resonance image studies.

### Statistical analysis

Local failure was defined as recurrence of the treated lesion, intrahepatic recurrence was defined as recurrence within the liver outside of the treated lesion, and extrahepatic metastasis was defined as recurrent disease at any site outside of the liver. Overall and progression-free survivals were estimated from the date of the start of SBRT to the date of a patient’s death, the last follow-up examination, or to the date of tumor recurrence. Categorical variables for the normalized and non-normalized groups were compared using the χ^2^-test, whereas continuous variables were compared using Student's *t*-test. The probability of cumulative survival was calculated using the Kaplan-Meier method and was compared using the log-rank test. Univariate and multivariate analysis was performed via the Cox proportional hazards model. A *p*-value < 0.05 was considered statistically significant. To reduce potential confounding effects in this retrospective study, propensity score based matching analysis was performed. All the possible clinical variables (age, gender, Eastern Cooperative Oncology Group performance status, Child-Pugh classification, viral etiology, tumor size, initial AFP, alanine aminotransferase, albumin, bilirubin, international normalized ratio, and radiation dose) were included in the propensity score matching. We performed caliper matching on the PS (nearest available matching). Pairs (normalized and non-normalized groups) on the PS logit were matched to within a range of 0.2 multiplied by the standard deviation [20]. The balance of covariates was measured by their standardized differences. A difference > 20 % of the absolute value was considered significantly imbalanced. All statistical analyses were performed using the SPSS statistical package (version 18.0; SPSS Inc., Chicago, IL, USA) and R software version 2.13 (R Foundation for Statistical Computing, Vienna, Austria; www.r-project.org). The R package of MatchIt was used for the propensity score analysis [21].

## Results

### Patient characteristics and AFP response

The characteristics of the 77 patients in our study are summarized in Table 1 according to normalization of AFP level. The median age was 60 years (range, 39–78 years) and 75.3 % were males. Most patients had an Eastern Cooperative Oncology Group performance status of 0 (54.5 %) or 1 (40.3 %) and were chronic carriers of the hepatitis B virus (74.0 %). Thirty-seven (48.1 %) of the 77 patients showed AFP normalization (normalized group). In the other 40 patients (51.9 %), AFP levels did not decrease below 20 ng/mL (non-normalized group). Median follow-up time was 22.2 months (range, 1.8–68.6 months). Among 77 patients, 73 underwent a variety of previous treatment. The types of treatments were as follows: 33 transarterial chemoembolization (TACE) only; 27 TACE and RFA; 2 TACE and percutaneous ethanol injection (PEI); 1 TACE, RFA, and PEI; 7 Resection and TACE; 2 Resection, TACE, and RFA; and 1 RFA only. The median number of previous treatment sessions was 4 (0–17) and the median period from the initial diagnosis of HCC to the start of RT was 14.9 months (0.2–176.3 months).

**Table 1:** Patient characteristics

Variables	All patients (n = 77)			Propensity score matched patients (50 pairs)		
	Normalized group	Non-normalized group	*P*	Normalized group	Non-normalized group	*p*
	(n = 37)	(n = 40)		(n = 25)	(n = 25)	
	No. of patients (%)	No. of patients (%)		No. of patients (%)	No. of patients (%)	
Age (years)			0.279			0.929
Median	62	59		58	60	
Range	47–78	39–78		50–69	39–78	
Gender			0.550			0.747
Male	29 (78.4)	29 (72.5)		19 (76.0)	18 (72.0)	
Female	8 (21.6)	11 (27.5)		6 (24.0)	7 (28.0)	
ECOG PS			0.197			0.156
0	23 (62.2)	19 (47.5)		16 (64.0)	11 (44.0)	
1–2	14 (37.8)	21 (52.5)		9 (36.0)	14 (56.0)	
Child-Pugh classification			0.945			0.480
A	28 (75.7)	30 (75.0)		19 (76.0)	21 (84.0)	
B	9 (24.3)	10 (25.0)		6 (24.0)	4 (16.0)	
Viral etiology			0.402			1.000
HBs-Ag (+)	29 (78.4)	28 (70.0)		20 (80.0)	20 (80.0)	
HBs-Ag (−)	8 (21.6)	12 (30.0)		5 (20.0)	5 (20.0)	
Tumor size (cm)			0.488			0.344
Median	2.0	1.7		2.1	1.6	
Range	0.8–4.5	1.0–4.4		1.0–4.5	1.2–3.6	
Initial AFP (ng/mL)			0.187			0.655
Median	48.6	128.0		50.2	113.0	
Range	20.8–1870.0	20.3–3250.0		21.2–1870.0	20.3–2490.0	
ALT			0.093			0.981
Median	25	28.5		27	25	
Range	9–55	11–185		9–55	14–116	
Albumin			0.383			0.395
Median	3.6	3.4		3.4	3.6	
Range	2.4–4.5	2.4–4.7		2.4–4.5	2.9–4.7	
Bilirubin			0.841			0.804
Median	1.1	1.1		1.0	1.0	
Range	0.5–3.5	0.6–6.2		0.5–2.9	0.6–6.2	
INR			0.550			0.514
Median	1.1	1.1		1.2	1.1	
Range	0.9–1.5	0.9–1.7		0.9–1.5	0.9–1.7	
Radiation dose			0.909			0.743
Median	45	45		45	45	
Range	36–60	36–60		36–60	36–60	

*Abbreviations*: *ECOG PS* Eastern Cooperative Oncology Group performance status, *HBs-Ag* hepatitis B surface-antigen, *AFP* alpha-fetoprotein, *ALT* alanine aminotransferase, *INR* international normalized ratio

Although baseline characteristics did not differ significantly between the two groups, propensity score matching was also performed. After this analysis, 25 matched pairs of the AFP normalized group vs. the non-normalized group were created. In this matched cohort, the baseline characteristics were distributed evenly between the two groups (Table 1).

### AFP response correlated to survival

**Fig. 1:**
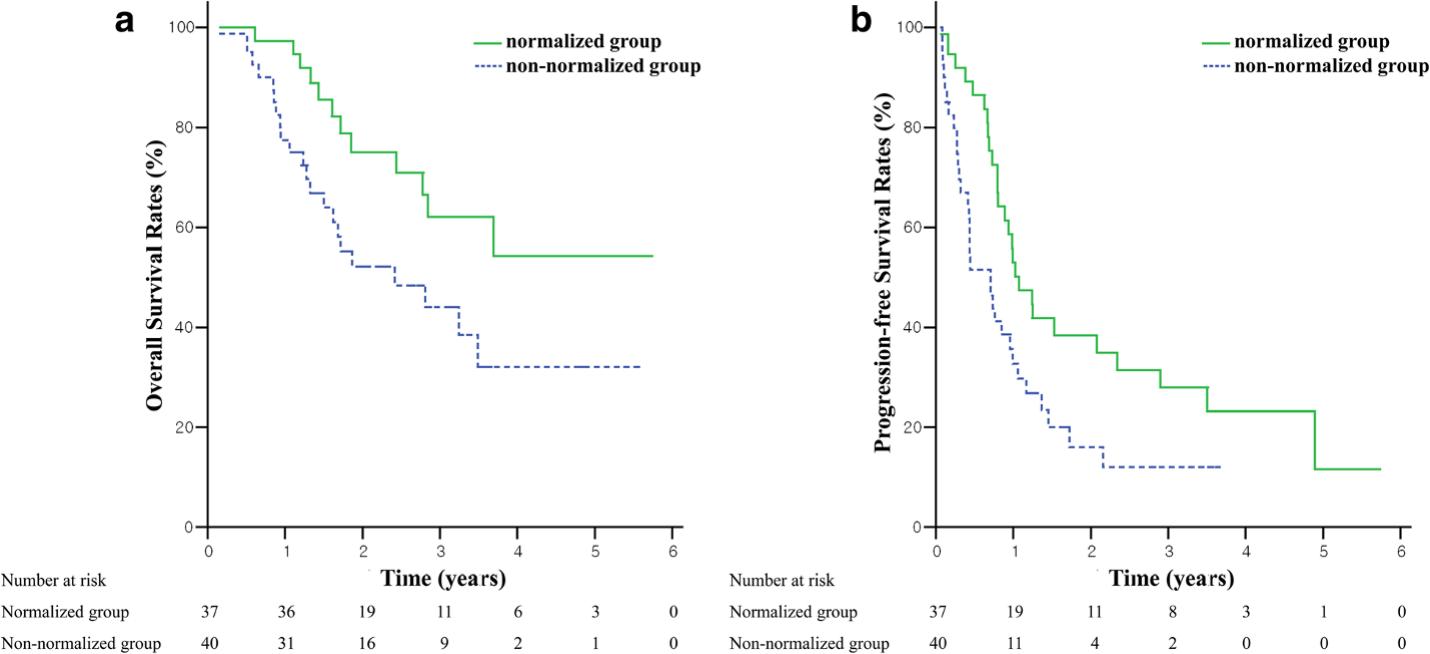
Overall survival (**a**) and progression-free survival rates (**b**) of all patients according to alpha-fetoprotein normalization

**Fig. 2:**
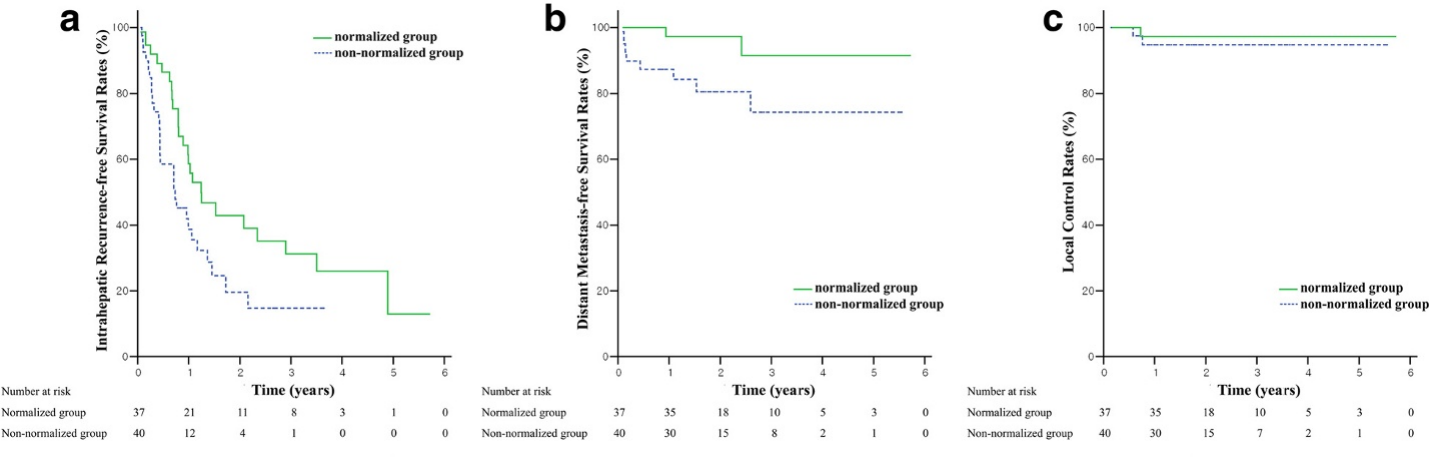
Intrahepatic recurrence-free survival (**a**), distant metastasis-free survival (**b**), and local control rates (**c**) of all patients according to alpha-fetoprotein normalization

The median OS and PFS time for all patients were 38.9 months and 10.7 months, respectively. OS at 3-year was superior in the normalized group to that in the non-normalized group (62.0 % vs. 44.0 %, *p* = 0.023). PFS at 3-year was also superior in the normalized group to that in the non-normalized group (27.9 % vs. 12.0 %, *p* = 0.019) (Fig. 1). Intrahepatic recurrence-free survival and distant metastasis-free survival were significantly longer in the normalized group than in the non-normalized group (*p* = 0.033 and 0.044, respectively). However, local control rates did not differ significantly between these two groups (97.2 % in the normalized group and 94.7 % in the non-normalized group at 3-year, *p* = 0.579) (Fig. 2).

**Fig. 3:**
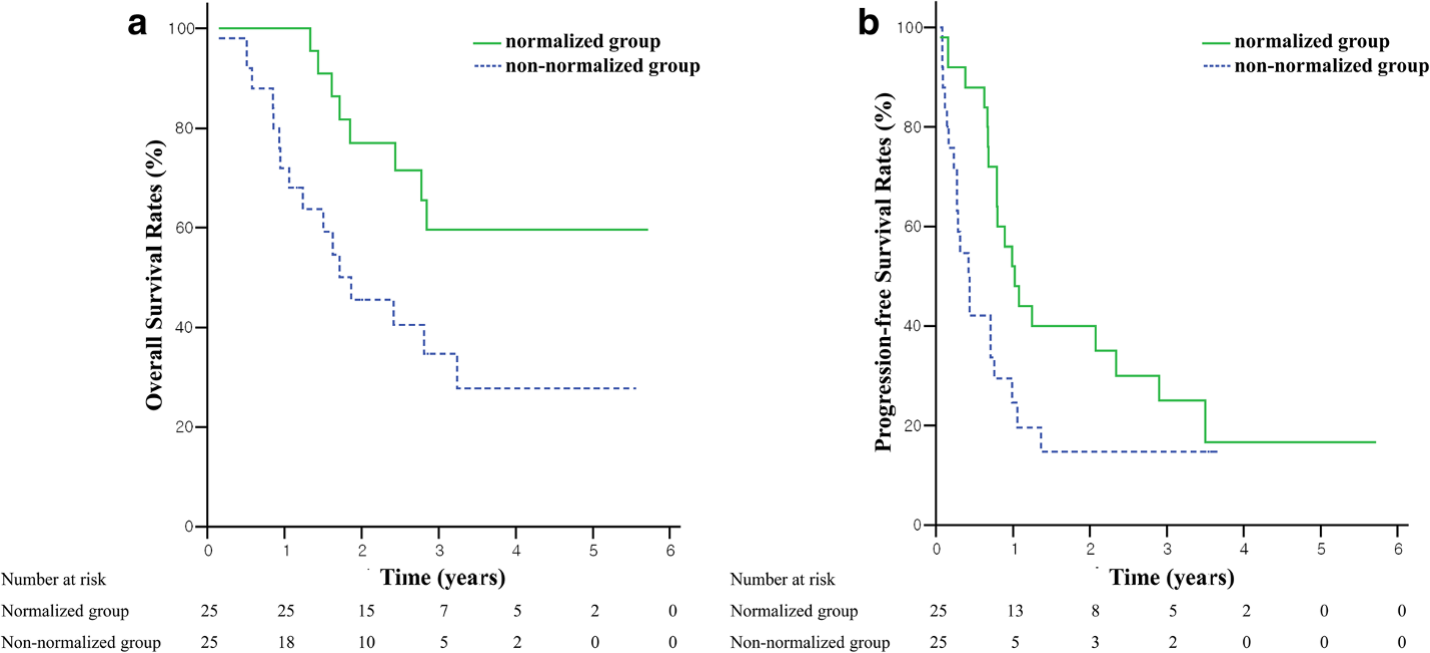
Overall survival (**a**) and progression-free survival rates (**b**) according to alpha-fetoprotein normalization in the propensity score matched cohorts

After propensity score matching, OS and PFS were still significantly longer in the normalized group than in the non-normalized group (*p* = 0.017 and 0.049, respectively) (Fig. 3). Intrahepatic recurrence-free survival and distant metastasis-free survival showed trends of longer survival in the normalized group (*p* = 0.114 and 0.068). The local control rate was 100 % in 50 of the matched cohort patients.

### Independent prognostic significance of AFP normalization

The univariate and multivariate analyses are summarized in Table 2. On univariate analysis, age, Child-Pugh classification, tumor size, albumin, bilirubin, radiation dose, and AFP normalization were identified as significant parameters of OS. Multivariate analyses revealed that the following factors had significant effects on OS: AFP normalization (hazard ratio [HR] = 0.31; 95 % CI, 0.14–0.70; *p* < 0.005); age (HR = 1.08; 95 % CI, 1.03–1.14; *p* < 0.004); and tumor size (HR = 1.66; 95 % CI, 1.14–2.40; *p* < 0.008).

**Table 2:** Univariate and multivariate analyses of prognostic parameters for overall survival

Variables	Univariate Analyses			Mutivariate Analyses		
	P (Cox regression)	HR	95 % CI	P (Cox regression)	HR	95 % CI
Age	0.035	1.05	1.01-1.09	0.004	1.08	1.03-1.14
Gender	0.654	1.19	0.56-2.55	-	-	-
ECOG PS (0 *vs.* 1–2)	0.129	1.72	0.85-3.46	-	-	-
Child-Pugh (A *vs.* B)	0.011	2.52	1.24-5.13	0.142	2.72	0.72-10.31
Tumor size (cm)	0.016	1.52	1.08-2.15	0.008	1.66	1.14-2.40
preAFP (ng/mL)	0.436	1.00	1.00-1.00	-	-	-
ALT	0.898	1.01	0.99-1.01	-	-	-
Albumin	0.050	0.53	0.28-1.00	0.379	1.62	0.55-4.72
Bilirubin	0.027	1.58	1.05-2.36	0.258	1.43	0.77-2.70
INR	0.065	7.99	0.88-72.87	0.400	4.48	0.14-146.79
Radiation dose	0.018	0.94	0.89-0.99	0.079	0.95	0.90-1.01
AFP normalization (yes *vs.* no)	0.028	0.45	0.22-0.92	0.005	0.31	0.14-0.70

*Abbreviations*: *HR* hazard ratio, *CI* confidence interval, *ECOG* Eastern Cooperative Oncology Group performance status, *HBs-Ag* hepatitis B surface-antigen, *preAFP* pre-treatment alpha-fetoprotein, *ALT* alanine aminotransferase, *INR* international normalized ratio

AFP normalization also had a significant prognostic value regarding OS and PFS after propensity score matching (Table 3).

**Table 3:** Hazard ratios for clinical outcomes according to AFP normalization in propensity score matched cohorts

Outcome (n = 50)	HR* (95 % CI)	*p-*value
OS	2.823 (1.205-6.612)	0.017
PFS	1.894 (1.002-3.578)	0.049

*Abbreviations*: *HR* hazard ratio, *CI* confidence interval, *OS* overall survival, *PFS* progression-free survival

*HRs for the AFP non-normalized group compared with the AFP normalized group

## Discussion

AFP has been reported to be a useful tumor marker for the detection of recurrence, and a predictive factor for patient survival after locoregional or systemic treatment in HCC [14–17]. Shirabe et al. demonstrated that a decline in AFP levels after surgery is a good predictive factor for the detection of early recurrence in patients with HCC [14]. One study showed a favorable decrease of AFP level after RFA as a prognostic surrogate for predicting the disease-free survival in HCC [17]. The prognostic value of AFP was also reported in studies on systemic therapy as well as in those on local therapy. In the study of Chan et al., the achievement of AFP response was an independent prognostic factor for patient survival, and serial AFP measurements were useful in monitoring treatment response in patients with unresectable HCC undergoing systemic chemotherapy [15]. Yau et al. also showed that a drop in serum AFP >20 % during the first six weeks is an exploratory early surrogate for both clinical benefit (i.e., complete response, partial response, or stable disease according to the Response Evaluation Criteria in Solid Tumors (RECIST) criteria) and PFS in advanced HCC patients receiving sorafenib [22].

The significance of the AFP response after external beam radiotherapy for HCC patients has not been fully evaluated. Kim et al. evaluated early AFP response as a predictor of clinical outcome after localized concurrent chemoradiotherapy (CCRT) for 149 advanced HCC patients [23]. This study demonstrated that OS and PFS were longer in AFP responders after CCRT. Lee et al. reported that AFP responders (which were defined as those with a reduction of > 20 % from the baseline level) showed significantly better OS after hepatic artery infusion chemotherapy or CCRT than did non-responders [24]. Other authors also reported association between AFP level and local tumor control or tumor response after SBRT for HCC [18, 19]. However, there has been no study to evaluate the significance of AFP normalization in patients with small HCC after ablative radiotherapy, especially SBRT.

In the present study, AFP normalization in patients with HCC after SBRT was a good prognostic surrogate for OS and PFS. Our data suggest that patients who achieve AFP normalization after SBRT have longer OS and PFS than do non-normalized patients. The difference in PFS between these two groups was mainly due to differences in incidence and the proportion of intrahepatic recurrence, and next by the difference in distant metastasis, while the local control rate was not significantly different between the normalized and the non-normalized group. OS and PFS were still longer in the AFP normalized group than in the non-normalized group after propensity score matching. We can assume that the difference in PFS affected OS, and that non-normalized AFP levels reflect the subclinical intrahepatic or extrahepatic tumor burden, which may be responsible for early recurrence after treatment. Lee et al. proposed a similar hypothesis, namely that increased AFP values may be related to the presence of tumor invasiveness indices, such as vascular invasion, tumor differentiation, and intrahepatic metastases, and that differences in tumor invasiveness may have occurred according to AFP response. If so, this could result in a difference in OS between AFP responders and non-responders [24].

AFP normalization is a simple, rapid, reproducible, and operator-independent measurement [16] and has been shown to be a prognostic surrogate. However, studies on the prognostic value of AFP normalization after SBRT are difficult due to several factors. First, there is as of yet no consensus regarding the optimal decrement of the AFP level. Furthermore, the cut-off level of pre-treatment AFP that would be adequate in order to apply AFP normalization as a surrogate is indistinct. Nevertheless, in our study we adopted a pre-treatment AFP level > 20 ng/mL as an inclusion criterion. Almost all of the patients who were analyzed in this study had an ALT level within the normal range, and most patients were chronic carriers of hepatitis B virus, i.e., they had no replicate activity. As patients with a high viral load were treated with an antiviral agent, they were in a stable state under viral suppression. Therefore, we could avoid disturbances caused by chronic hepatitis. However, when clinicians apply AFP normalization as a prognosticator, they should be cautious regarding the liver condition of their patients. Second, interpretation of AFP normalization could be ambiguous because of variable patient and tumor characteristics. In our study, all patients were treated with SBRT for small viable HCC as the salvage treatment intent after undergoing several previous severe treatments, namely transarterial chemoembolization, hepatectomy, and/or RFA. These states may be related to more frequent progression or intrahepatic or extrahepatic recurrence than is seen in treatment-naïve patients who were enrolled in previous studies and who demonstrated significant post-treatment AFP after surgery or RFA. Third, the value of AFP normalization in our study can only be applied in patients who have elevated AFP levels before treatment. Future studies regarding serum protein induced by vitamin K absence or antagonist-II (PIVKA-II) could reveal the usefulness of this tumor marker for HCC patients with a normal AFP level [24]. Finally, although this study was performed using 20 ng/mL as the cut-off level for elevated AFP, among 165 patients who underwent SBRT, only 77 patients were included in our study. Furthermore, the sample size was further decreased after propensity score matching analysis. Therefore, further studies with a greater number of patients could provide a more relevant answer regarding AFP normalization.

Despite these limitations, ours is the first study showing the value of AFP normalization as a prognostic surrogate in OS and PFS in two cohorts that were evenly distributed through propensity score matching analysis. AFP normalization seems to be a useful tool for predicting clinical outcomes in small HCC after local ablative therapy.

## Conclusions

AFP normalization in patients with small HCC within three months after SBRT is a prognostic surrogate for OS and PFS. Patients who achieve AFP normalization after SBRT have longer OS and PFS than do those in a non-normalized group. AFP monitoring should be considered a useful tool in HCC patients with an elevated AFP level before SBRT.

## Abbreviations

AFP: alpha-fetoprotein
SBRT: stereotactic body radiotherapy
HCC: hepatocellular carcinoma
OS: overall survival
PFS: progression-free survival
RFA: radiofrequency ablation
CT: computed tomography
GTV: gross tumor volume
ITV: internal target volume
HR: hazard ratio
RECIST: Response Evaluation Criteria in Solid Tumors
CCRT: concurrent chemoradiotherapy
PIVKA-II: protein induced by vitamin K absence or antagonist-II
